# Customization of Diet May Promote Exercise and Improve Mental Wellbeing in Mature Adults: The Role of Exercise as a Mediator

**DOI:** 10.3390/jpm11050435

**Published:** 2021-05-19

**Authors:** Lina Begdache, Cara M. Patrissy

**Affiliations:** 1 Health and Wellness Studies Department, Binghamton University, Binghamton, NY 13890, USA; 2 Department of Psychology, Integrative Neuroscience, Binghamton University, Binghamton, NY 13890, USA; cpatris1@binghamton.edu

**Keywords:** diet, dietary practices, mental health, exercise, customization, gender, adults, brain maturity

## Abstract

Diet, dietary practices and exercise are modifiable risk factors for individuals living with mental distress. However, these relationships are intricate and multilayered in such a way that individual factors may influence mental health differently when combined within a pattern. Additionally, two important factors that need to be considered are gender and level of brain maturity. Therefore, it is essential to assess these modifiable risk factors based on gender and age group. The purpose of the study was to explore the combined and individual relationships between food groups, dietary practices and exercise to appreciate their association with mental distress in mature men and women. Adults 30 years and older were invited to complete the food–mood questionnaire. The anonymous questionnaire link was circulated on several social media platforms. A multi-analyses approach was used. A combination of data mining techniques, namely, a mediation regression analysis, the K-means clustering and principal component analysis as well as Spearman’s rank–order correlation were used to explore these research questions. The results suggest that women’s mental health has a higher association with dietary factors than men. Mental distress and exercise frequency were associated with different dietary and lifestyle patterns, which support the concept of customizing diet and lifestyle factors to improve mental wellbeing.

## 1. Introduction

Interdisciplinary research on mental distress and lifestyle suggests that diet, dietary practices and exercise are modifiable risk factors that are associated with mental wellbeing [1]. However, these relationships are complicated and multidimensional. One important dimension that needs to be considered is the effect of gender on these risk factors. Increasing evidence indicates that there is a disparity in the prevalence of mental distress among men and women [2]. Imaging studies describe the morphological differences in the gender brain [3,4,5,6] and propose a connection with brain functionality [7]. These structural variations may require a differential repertoire of nutrients and lifestyle factors for optimal brain function. In addition, taking into consideration the age bracket based on adult brain development is another layer of importance. Typically, the brain completes its development between the mid–late 20s [8], and post-maturity is typically linked to slow brain aging. Therefore, diet quality and lifestyle factors may have a different impact on the mature brain when compared to its younger counterpart. Dietary practices, such as eating breakfast and use of dietary supplements, may improve brain health and boost mental wellbeing through a heightened motivation to lead a healthy lifestyle.

Exercise supports the release of a number of growth and neurotrophic factors that are associated with neuroplasticity [9]. Precisely, the vascular endothelial growth factor (VEGF), an angiogenic signaling molecule, improves age-related changes in cerebral blood flow and capillary vascularity [10]. The brain-derived neurotrophic factor (BDNF) promotes neurogenesis and neuroplasticity [11], while the insulin-like growth factor (IGF-1) confers neuroprotection and neural repair [12]. Therefore, exercise supports processes that improve cognitive functions and mental wellbeing, which are essential for the matured brain [13]. Several studies described individually the impact of diet and exercise on mental wellbeing; however, the integrated associations between dietary patterns, dietary practices and exercise frequency on mental distress in mature adults have not been explored. Therefore, through this study, we wished to identify the individual and combined relationships between these variables and their implication on mental distress among mature men and women. Results from this study could provide the framework needed for customizing dietary plans to promote exercise and improve mental wellbeing in mature adults. They could also provide a new perspective for the research community when assessing the role of diet on mental distress. 

## 2. Methods

### 2.1. Participants

This study is part of a larger project that has been collecting data to investigate dietary patterns and mental distress in different cohorts. The anonymous questionnaire link was distributed on several social media platforms targeting social and professional groups. Participants consented to the study by agreeing to access the survey. The larger study collected data from all age groups; however, for the purpose of the current study, only data from adults 30 years or older were considered. The study protocol was reviewed and approved by the Institutional Internal Review Board. The only inclusion criteria used were healthy adults and over the age of 18 years. No pre-screening of mental health was performed beforehand, as the purpose was to assess the relationship between diet and different levels of mental distress. 

A minimum sample size with 95% confidence and a 5% margin of error for an estimated population size of 2000 was set at 322 samples for each gender. Data collection was performed over a 3-year period and at different intervals to account for the change in season as well as to diversify the target population. 

### 2.2. Study Design

The Food–Mood Questionnaire (FMQ) was used for dietary and mood data collection [14]. Demographic questions included gender, age groups (the ones included in the study were: 30–39, 40–49, 50 and above), region of residence, highest education achieved (high school, 2–4 years of college AA, BS, BA), and the type of dietary patterns followed. The food groups assessed were whole grain, fruits, dark green leafy vegetables (DGLV), meat (white and red), beans and legumes, nuts, dairy, fish and high glycemic index (HGI) foods. Frequency of breakfast consumption and exercise, use of multivitamins and fish oil supplements, consumption of fast food and caffeinated beverages were also assessed. The question on exercise asked about the frequency of exercising (cardiovascular or strength exercise) for at least 20 min a day. Evidence from the literature suggests that at least 20 min of exercise a day, regardless of the type, improves mental wellbeing [15,16,17]. 

The FMQ is a validated instrument that evaluates weekly servings of food groups known to influence brain function and chemistry. The FMQ is a 5-subscale item with an internal consistency, as reflected by Cronbach’s alpha values ≥ 0.70 for all sub-scales. The FMQ is a reliable tool (Intraclass Class Coefficient 0.619–0.884; *p* < 0.01; CI 95%). The FMQ also has an external validity [14]. FMQ questions use a 6-point Likert scale and consist of six questions on mental distress adopted from the Kessler-6 psychological distress scale (K-6) [18]. The K-6 scale is a quantifier of a spectrum of mood, and its questions originate from item response theory. The K-6 has consistent psychometric properties across major socio-demographic sub-samples and strongly distinguishes between community cases and non-cases of DSM-IV/SCID [19]. Therefore, the total sum of the K-6 was used to assess mental distress. 

### 2.3. Classification of Dietary Patterns

The healthy dietary pattern was classified based on the recommendations by the Dietary Guidelines for Americans 2020–2025, which include a spectrum of nutrient-dense food such as fruits, vegetables, whole grains, beans, nuts, lean meat and low-fat dairy [20]. The unhealthy dietary pattern was classified based on the USDA published results of the 1994 Continuing Survey of Food Intakes by Individuals (CSFII), describing the standard American diet [21], which includes dairy, meat, high glycemic index food and fast food and excludes fruits, vegetables, legumes and fish consumption. Dietary practices were established around the clustering of breakfast and/or multivitamin and fish oil supplements use. Other non-classical categories of patterns were based on food groups with high consumption.

### 2.4. Data Partitioning

Adults 30 years or older were classified as mature adults (MA), as suggested in the literature [8,22]. To categorize the degree of mental distress among participants, the K-6 sum scores were characterized into three levels: low mental distress (0–7), moderate (8–12), and high (13 and above) [23], which have been used in the literature [19]. 

### 2.5. Statistical Analysis

#### 2.5.1. Data Pre-Processing

Data cleaning took place in Google sheets using the data cleaning option to remove duplicate entries. Data values were standardized into z scores. 

#### 2.5.2. Rationale of the Study Design

**Figure 1:**
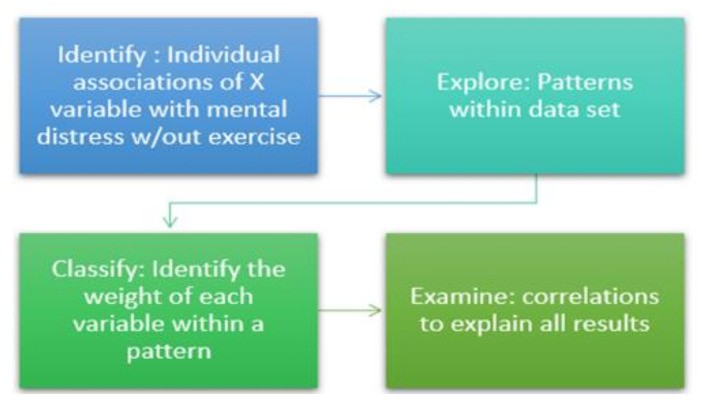
Study design rationale.

We used a multi-analyses approach to investigate the individual and combined relationships between dietary factors, dietary practices, exercise and their implication on mental distress among mature men and women. The first step was to perform a mediation regression analysis (MA) to identify: (1) the individual variables that are associated with mental distress, (2) the individual variables that are linked with exercise frequency and (3) the association of each independent variable on mental distress when exercise is a mediator. Next, a K-means cluster analysis (CA) was used to classify the dataset into clusters to extrapolate unidentified patterns within the dataset and with no prior labeling. K-means is an unsupervised machine learning technique that detects hidden patterns without human guidance. This step explained the MA results and further explored the direct and inverse associations between food groups, dietary practices and exercise with mental distress. A principal component analysis (PCA) was employed as a confirmatory method to validate the results obtained from MA and CA. The PCA reduces data dimensionality and identifies patterns within the dataset, while providing a loading factor that represents the weight of the variable within the pattern. This attribute clarified the potential contribution of each variable within a dietary pattern to mental status. The fourth step was to perform a two-tailed correlational study to further explore the relationships between the independent variables, exercise and mental distress and confirm the findings from previous analyses (Figure 1). 

#### 2.5.3. Mediation Analysis

**Figure 2:**
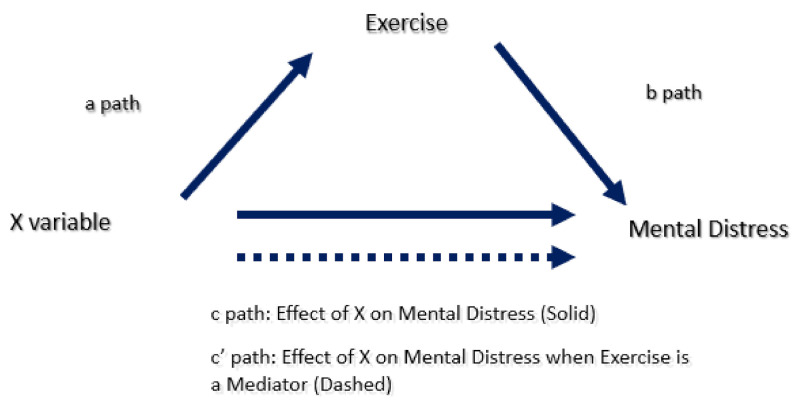
The mediation regression model.

The mediation analysis used PROCESS Macro version 3.0, model four [24], with a 5000-bootstrapping sampling option and a 95% confidence interval. The mediation analysis assessed three different effects or paths. Path “a” defined the regression coefficients for the association of the independent variable (X) on exercise. Path “b” explored the link between exercise and mental distress. Pathway “c” assessed the direct effect of X on mental distress, while pathway “c’” estimated the mediation coefficient (or indirect effect) of exercise on X in relation to mental distress (Figure 2). The mediation effect is significant if CI excludes a zero. Thus, if it includes a zero, it means that running the analysis again suggests no correlation.

#### 2.5.4. Cluster Analysis

The K-means cluster analysis was an investigative step to produce different dietary, exercise and mental distress clusters. Standardization of variables as z-scores gave them an equal weight and minimized the influence of the outliers. The K-means clustering algorithm used iteration to partition the dataset into a pre-specified number of k distinct clusters. Each training instance was allocated to the closest centroid based on the Euclidean distance applied to the instance and cluster center. All centroids were then recalculated as the mean attribute value vectors of the instances that were assigned to specific clusters. The cluster centers were adjusted by randomly picking k training instances, where k was the assigned number of clusters. If the cluster centroids remained constant, the iterative process stopped. The K-means analysis was followed by an ANOVA analysis to confirm the significance of each variable within the clusters. 

#### 2.5.5. Principal Component Analysis

The PCA analysis identified the different patterns within the dataset with a component loading. Data were stratified by gender and further by principal components (PC). Sampling adequacy and inter-correlation of variables were calculated using the Kaiser-Meyer-Olkin (KMO) test and Bartlett’s test of sphericity, respectively. The eigenvalue ≥1.0 criterion was used to determine the number of PCs retained. Additionally, the number of PCs selected was confirmed by visually examining the first major infliction in the scree plot. The optimal number of components typically capture the highest amount of variance in the dataset. Using varimax rotation, PCs were orthogonally rotated (varimax) to simplify and enhance their interpretability [25]. Variables with component loading (CL) of ≥0.2 were considered significant contributors to the patterns and were included in the PC solution [26]. Positive and negative loadings suggested direct and inverse relationships with the PCs, respectively. Components were classified into healthy diet, unhealthy diet and supplements use PCs. A one-way ANOVA was used to compare the means of the dietary patterns.

#### 2.5.6. Spearman’s Rank–Order Analysis

An assessment of data normality using Shapiro–Wilk and Kolmogorov–Smirnov tests suggested that the data are not normally distributed. Subsequently, a two-tailed Spearman’s ank–order correlation evaluated the strength and direction of the relationship between the variables of interest. Data analysis was performed using SPSS version 25.0. 

## 3. Results

A total of 1209 records were analyzed from mature adults (30 years or older); 329 were from mature men, 880 were from mature women. Responses were collected from North America, Europe, the Middle East and North Africa (MENA). Participants’ characteristics are described in Table 1. 

### 3.1. Mediation Analysis

The mediation analysis provided three significant sets of findings. (1) It identified the direct association of food groups and dietary practices on mental distress (c path). (2) It explained the effect of each of these independent variables on mental distress when exercise is a mediator (c’ path). (3) It described the association between the independent variables and exercise frequency (a path). Since the b path always compared the association of exercise on mental distress, the results were similar for all generated models. 

#### 3.1.1. The c and c’ Paths

Results of the mediation analysis are presented in Table 2. Among women, there was a significant association between fast food (b = 0.4186) and mental distress. And a moderate association between caffeine (b = 0.2371), HGI food (b = 0.344) and metal distress. In addition, there was a moderate negative association between breakfast (b = −0. 3819), fruits (b = −0.2532), DGLV (b = −0.3511), fish (b = −0.2694) and mental distress (c path).

Exercise significantly reduced the negative association of HGI food and fast food on mental distress. Interestingly, exercise reversed the negative outcome of caffeine on mental distress and it significantly improved the positive aspect of breakfast, fruits, DGLV, fish and mental stress. Exercise, as a mediator, also generated novel inverse associations between food groups such as whole grain, nuts, multivitamin and fish oil supplements and mental distress. Remarkably, exercise as a mediator produced a positive association between meat and mental distress (c’ path). 

Among men, no dietary factors were positively associated with mental distress. There was a strong significant and inverse relationship between nuts (b = −0.4347), fish (b = −0.5430) and mental distress (c path). Exercise improved the positive outcome of nuts and fish on mental wellbeing. Exercise, as a mediator, also generated novel inverse associations between breakfast, whole grain, dairy, fruits, DGLV, beans, multivitamin and fish oil supplements and mental distress. Exercise did not have any significant relationship with caffeine and meat regarding mental wellbeing. A noteworthy observation was that although HGI food and fast food were significantly associated with mental distress, exercise as a mediator produced a significant positive relation with mental distress (c’ path).

#### 3.1.2. The a Path

Among women, factors that associated with exercise included breakfast (b = 0.2270), caffeine (b = 0.0652), whole grain (b = 0.1927), fruits (b = 0.1420), nuts (b = 0.2031), DGLV (b = 0.3329), beans (b = 0.1783), fish (b = 0.3926) and MV (b = 0.0725) and fish oil supplements (b = 0.1293). Those that are inversely associated with exercise included HGI food (b = −0.1829), meat (b = −0.0861) and fast food (b = −0.3646). Dairy was not significantly associated with exercise. In men, HGI (b = −0.2044) and fast food (b = −0.2168) had an inverse association with exercise. Factors that strongly correlated with exercise included breakfast (b = 0.4550), whole grain (b = 0.2651), dairy (b = 0.1782), fruits (b = 0.2614), nuts (b = 0.3096), DGLV (b = 0.2773), beans (b = 0.1746), fish (b = 0.3753) and MV (b = 0.1544) and fish oil (b = 0.2569) supplements. Meat was not significantly associated with exercise (Table 2).

#### 3.1.3. Cluster Analysis

The cluster analysis identified two dietary patterns (DP) and three DP for mature men and women, respectively. The significant variables for women in Cluster 1 consisted of a healthy dietary pattern and practices. It included exercise, breakfast, whole grain, dairy, caffeine, fruits, nuts, HGI food, meat, DGLV, beans, fish, fish oil and an inverse association with mental distress (mean value = −0.11843). Cluster 2 was a Western diet pattern and consisted of HGI food, meat and mental distress (mean value = 0.64137). Cluster 3 included mostly dietary practices, such as breakfast, fish, MV and fish oil, exercise and mental wellbeing (mean value = −0.31067). Among men, Cluster 1 included fast food and mental distress (mean value = 0.16357), which represented a Western diet pattern. Cluster 2 reflected all food groups including nuts and fish and excluded fast food, dietary practices and exercise. This pattern is associated with mental wellbeing (mean value = −0.22241) (Table 3).

#### 3.1.4. PCA

The PCA revealed additional interesting findings. There were three principal components (PCs) identified for men and women with a total variance of 37.898% and 38.616%, respectively. Among women, PC 1 consisted of some exercise (CL = 0.241), a healthy diet and dietary practice. This pattern included triggers of mental distress such as caffeine (CL = 0.656) and HGI food (CL = 0.212) as well as impediments to mental distress such as fruits (CL = 0.77), breakfast (CL = 0.31) and DGLV (CL = 0.368). Interestingly no negative or positive CL was noted for mental distress. This PC explained 17.477% of the total variance. PC 2 was mostly supplement use with exercise (CL = 0.304). This pattern seemed to draw protein intake mostly from beans (CL = 0.677) and about half from fish (CL = 0.311), with no animal proteins included. Interestingly, no negative or positive CL were also noted for mental distress. This PC explained 10.775% of the total variance. PC3 was close to the Western diet, which included strong loadings for HGI food (CL = 0.685), fast food (CL = 0.583) and mental distress (CL = 0.523). This pattern excluded exercise, DGLV and fish. This PC explained 10.364% of the total variance.

Among men, PC1 reflected a healthy dietary pattern, exercise (CL = 0.388) and a negative association with mental distress (CL = −0.214). This PC explained 14.812% of the total variance. PC2 was a dietary practices pattern with some healthy food groups and exercise (CL = 0.509). Interestingly, this pattern was also negatively associated with mental distress (CL = −0.288). This PC explained 13.968% of the total variance. PC3 was close to the Western diet which included strong loadings for meat (CL = 0.695), HGI food (CL = 0.657), DGLV (CL = 0.418) and caffeine (CL = 0.383). Although this pattern is known to be associated with mental distress, no loading (positive or negative) surfaced. This PC explained 9.118% of the total variance (Table 4).

#### 3.1.5. Spearman’s Rank–Order Correlation

Results from Spearman’s correlation analysis are presented in Table 5; Table 6. Among women, exercise was positively correlated with breakfast, whole grain, fruits, DGLV, beans, MV and fish oil supplements (*p* < 0.001). There was an inverse association between exercise, HGI and fast food and mental distress (*p* < 0.001). Mental distress positively correlated with HGI and fast food (*p* < 0.001) as well as caffeine (*p* < 0.05), and inversely associated with exercise, breakfast, fruits, DGLV and fish (*p* < 0.001). There was a strong association between consumption of one healthy food group with most other food groups, and exercise was associated with eating a spectrum of nutrient-rich food. Among men, exercise was correlated with breakfast, whole grain, dairy, fruits, nuts and DGLV, fish, MV and fish oil. It was negatively associated with HGI and fast food (*p* < 0.001). Interestingly, mental distress was not linked with any food groups. However, factors that were inversely related to mental distress included exercise, fruits, nuts, fish and MV supplements. When comparing men and women, dairy was the factor that was strongly associated with exercise in men, but not in women (r = 0.161 **). For women, beans were a factor that significantly correlated with exercise, but was not significant in men (r = 0.119 **). 

## 4. Discussion

Several interesting findings were revealed in this study. Our results suggest that food groups within a dietary pattern and frequency of exercise have a differential relationship with the mental wellbeing of mature men and women. Additionally, mental distress in women is more likely to be impacted by dietary factors than men, which supports previous findings [27]. Our results also suggest that there is an association between food groups and exercise frequency. Several of these groups are low in energy but packed with micronutrients such as B vitamins and minerals as well as the healthy fats needed for neurotransmitter synthesis [28] and brain function [29,30], respectively. This finding proposes that further studies are needed to elucidate the physiological mechanisms of how exercise modulates the relationship of particular food groups with brain chemistry. The following section summarizes the major findings from each analysis. 

### 4.1. Mediation Regression Analysis (MA)

The individual relationship between exercise, food groups and dietary practices was explored through MA. In general, exercise enhanced the positive relationship of mental distress impediments and reduced the negative impression of mental distress triggers. Several studies described that exercise improves mental health significantly [9,10,31]; to our knowledge, this study is the first in proposing the potential enhancing effect of food groups on mental health when exercise is a mediator. One potential mechanism that may explain this phenomenon is the reciprocal relationship between exercise and food groups. The fact that several of these food groups were associated with higher exercise frequency suggests that there is a potential virtuous relationship between healthy food groups and exercise that eventually improves mental wellbeing. Healthy food groups are labeled as such because they confer several health benefits including complex carbohydrates, essential amino acids and healthy fats such as omega-3 fats. In addition, maintenance of a steady blood glucose (high fiber, low glycemic index food), normal blood pressure (food high in potassium and low in sodium) and strong skeletal muscle (complete proteins and omega-3 fats) support the ability to exercise. Omega-3 fats have an anti-inflammatory property that supports muscle integrity. These food groups were also all reported to have a positive association with mood [32,33,34]. Another interesting observation noted was that exercise reversed the negative relationship of caffeine on mental distress in women but did not reflect any association in men. From our findings, women in our cohort had mostly low levels of exercise. Caffeine appears to be a double-edged sword. As a stimulant, it activates the hypothalamic, pituitary, adrenal (HPA) axis that modulates the stress response [35]. As an adenosine antagonist, caffeine delays fatigue during exercise. Therefore, women who consume high levels of caffeine and do not exercise are more likely to experience mental distress. However, those who consume caffeine and exercise, are more likely to get the neuromodulator benefit of exercise by working out longer. This may explain the conflicting reports of the association between caffeine and mood that may have excluded exercise as a potential mediator [36,37]. In a few instances in our study, exercise acted as a trigger for mental distress with consumption of certain food groups. For women, meat did not have any association with mental distress, but became a trigger with exercise. The same applies for fast food and mental distress in men.

The relationship between meat, exercise and mental distress is intriguing. The conventional thought is that meat is a good source of tryptophan and tyrosine, the precursors for serotonin and dopamine, respectively. However, digging into the biochemistry of these neurotransmitters may reveal a response. The transport of tryptophan and tyrosine across the blood brain barrier is insulin-dependent [38,39]. Exercise typically lowers blood glucose, which improves the insulin response. In addition, women appear to have lower kinetics for serotonin production [40], which means slight alterations in the tryptophan influx to the brain may modulate serotonin synthesis. The relationship between fast food, exercise and mental distress in men is more of a straightforward connection. Typically, fast food is high in salt, sugar and saturated fat, and as a low-quality food, it is deficient in several nutrients. Exercise, being metabolically taxing, competes with the brain for essential nutrients. 

### 4.2. Cluster Analysis (CA)

The data mining techniques were used to explore the dynamic interaction between dietary patterns, dietary practices and exercise on mental distress. The CA confirmed many of the MA findings and generated new perspectives as well. For women, Cluster 1 was comprised of exercise, a healthy diet and healthy dietary practices pattern, which are associated with mental wellbeing. This is in line with several studies that investigated dietary patterns, practices, exercise and mental distress [37,38,39]. Cluster 2 was a Western diet pattern that is strongly associated with mental distress, which confirmed previous findings in the literature [41]. Cluster 3 described exercise and a healthy dietary pattern and an association with mental wellbeing. All three clusters confirmed the MA results in women. For men, Cluster 1 was comprised of fast food, which is reflective of a Western dietary pattern. This cluster was associated with mental distress; however, it was not justified by MA findings, which suggests that other confounding factors may have mediated this association between fast food and mental distress among mature men. Another potential factor is the duration of following this pattern. Nutrient imbalances are associated with mental distress [42]; however, since the study did not assess this component, it is suggested with caution as a potential contributor. In addition, fast food and the Western dietary pattern are typically associated with excessive saturated fat intake. The latter alters metabolism and exercise levels, which eventually lead to weight gain and subsequently to mental distress [31,43].

As for Cluster 2, it was comprised of a healthy diet, healthy dietary practices and exercise and, as expected, it was associated with mental wellbeing. Cluster 2 confirmed MA results as exercise significantly improved the association between these food groups with mental health. When comparing the results between gender, the Western dietary pattern produced a higher mean value for mental distress in women than in men. This finding suggests that mood among women has a stronger relationship with dietary factors when compared to men [27,44].

### 4.3. PCA

The usefulness of the PCA is in the component loading (CL) which provides a weight of the variable within the pattern. According to the MA results, exercise reversed the negative relationship of caffeine among women. However, looking at the component loading weight of caffeine and exercise in PC1, caffeine had about three times that of exercise in the healthy dietary pattern and yet, no loading for mental distress (negative or positive) was detected. This discrepancy suggests that the relationship between caffeine and exercise is not as straightforward as anticipated. In addition, it suggests the significance of the food group within a pattern and frequency of exercise play a role in mental health. Three general observations were noted from the PCA results based on the component loading values: (1) low to moderate exercise supported mental wellbeing among men regardless of the food groups consumed; (2) low exercise did not produce mental wellbeing in women despite the inclusion of healthy food groups and practices; and (3) when mature women consumed triggers of mental distress or relied on beans as a main source of proteins, low exercise did not chemically modulate enough of the mental distress impediments to produce mental wellbeing. This suggests that with more triggers of mental distress and a vegetarian style pattern, there is potentially a higher need for exercise to achieve mental wellbeing. The PCA results for men confirmed the MA findings that no dietary triggers seem to be associated with mental distress in men. The observations from the PCA also supported the notion that inclusion of impediments to mental distress such as nuts and fish support mental wellbeing. This differential relationship between exercise and mental wellbeing among men and women is interesting. Dissecting this finding further, the difference could be explained by the frequency or intensity of exercise [45] or could be the inherent difference in muscle mass [46]. This finding is worth further exploration. 

### 4.4. Spearman’s Rank–Order Correlation Analysis

Spearman’s correlation analysis confirmed the gender-based MA findings and revealed additional noteworthy conclusions. It confirmed the MA findings that triggers of mental distress for women were caffeine, HGI and fast food. Positive influencers of mental wellbeing were breakfast, fruits, DGLV and fish. The results also explained the association between HGI and fast food, low exercise and mental distress. They also proposed that consumption of healthy food groups may improve diet quality and frequency of exercise, which confirmed the findings from the MA. This evidence suggests that food groups may neurochemically promote the motivation to exercise. As for men, the results were comparable to the findings from the MA and corroborated that mental distress in men was not associated with any food group. However, Spearman’s correlation added fruits and MV supplements to the positive influencers of mental wellbeing in men, which explained some observations noted in the cluster analysis and PCA results. 

### 4.5. Summary of Findings

The most important take away points of this study are: (1) women are sensitive to the inclusion of triggers in their diet despite consumption of a healthy diet; (2) exercise (type and frequency) may have a differential relation with the mental wellbeing of men and women; (3) the weight of a dietary factor within a pattern may significantly sway mental wellbeing, which is more pronounced in women; and (4) research on diet and mood should take into consideration the potential indirect effect of exercise as a mediator. 

Another remarkable theory emerged for women: the quality of protein consumed may be important for women’s mental wellbeing, which requires further examination. Finally, our results suggest that despite following a healthy diet and lifestyle, if triggers of mental distress exceed certain thresholds, mood is negatively impacted.

### 4.6. Strengths and Limitations of the Study

The major strengths of the study are the large sample size and the use of a multiple- analyses approach to extricate the findings and illustrate the complex relationship between food groups, dietary patterns and practices, exercise and mental distress. The study fills several gaps in the literature by reporting on the differential influence of dietary factors and exercise on the mood of mature men and women. Furthermore, the results provide compelling evidence that the customization of diet and lifestyle may enhance mental wellbeing in this population. Finally, new theories emerged from this study that are worth further exploration. Nonetheless, the limitations of this study include the convenience sample and its cross-sectional design. In addition, it does not take into consideration the disparities in genetic factors, socio-economic status, sleep pattern, cultural differences or any other factors that may have impacted the psychology of the individuals or their food intake. 

## 5. Conclusions

Our study revealed several interesting findings. Among women, mood is more sensitive to dietary factors than men. Exercise may lessen the intensity of negative triggers, but it may not completely reverse it when frequency is low. The weight of the trigger also has an impact within the dietary pattern. For men, consumption of fast food and absence of exercise were associated with mental distress. However, low to moderate exercise seemed to significantly improve their mental wellbeing. In essence, our findings suggest that the customization of diet and exercise for mature men and women is needed to improve mental wellbeing. 

## Figures and Tables

**Table 1:** Participants Characteristics.

Category		Frequency	Percent
Gender			
	Males	329	27.2
	Females	880	72.8
Age			
	30–39	321	26.5
	40–49	426	35.3
	50–above	461	38.2
Region			
	Western countries	704	58.5
	MENA and Asian countries	499	41.5
Education			
	Less than high school	119	10
	High School	576	48.2
	2–4 years college	372	31.1
	Graduate degree	128	10.7

**Table 2:** Mediation regression analysis results for mature men and women.

		Men			Women		
Independent	Direct Effect of X on Mental Distress	Association between X and Mediator	Mediation or Indirect Effect	Direct Effect of X on Mental Distress	Association between X and Mediator	Mediation or Indirect Effect	
Variables (X)	C path (SE) *p*	A path (SE) *P*	C’ path (SE) CI	C path (SE) *P*	A path (SE) *p*	C’ path (SE) CI	
Caffeine	0.1468 0.1134 0.1965	0.0747 0.0576 0.1957	−0.0266 0.0233 [−0.0788–0.0133]	0.2371 0.0839 0.0048	0.0652 0.0331 0.0493	−0.0298 0.0170 [−0.0671–−0.0002]	
Breakfast	0.2222 0.2108 0.2926	0.4550 0.1024 0.0000	−0.1702 0.0657 [−0.3167–−0.0567]	−0.3819 0.1730 0.0275	0.2270 0.0626 0.003	−0.0943 0.0345 [−0.1719–−0.0340]	
Whole Grain	−0.0717 0.1062 0.5000	0.2651 0.0544 0.0000	−0.0869 0.0339 [−0.1611–−0.0275]	0.0372 0.0858 0.6649	0.1927 0.0340 <0.001	−0.0874 0.0230 [−0.1368–−0.0479]	
Dairy	−0.0253 0.1144 0.8252	0.1782 0.0578 0.0022	−0.0610 0.0293 [−0.1272–−0.0149]	0.1583 0.0874 0.0704	−0.0202 0.0340 0.5527	0.090 0.0156 [−0.0205–0.0412]	
Fruits	−0.1673 0.1202 0.1645	0.2614 0.0602 0.0000	−0.0809 0.0355 [−0.1602–−0.0212]	−0.2532 0.0921 0.0061	0.1420 0.0351 0.0001	−0.0581 0.0192 [−0.0999–−0.0253]	
Nuts	−0.4347 0.1122 0.0001	0.3096 0.0619 0.0000	−0.0765 0.0374 [−0.1594–−0.0116]	−0.0988 0.0877 0.2602	0.2031 0.0346 0.0000	−0.0859 0.0231 [−0.1356–−0.0459]	
HGI Food	0.1224 0.1335 0.3600	−0.2044 0.0662 0.0022	0.0676 0.0311 [0.0159–0.1385]	0.3444 0.1043 0.0010	−0.1828 0.0424 0.0000	0.0728 0.0231 [0.0330–0.1227]	
Meat	0.0559 0.1257 0.6569	0.1164 0.0702 0.0981	−0.0408 0.0286 [−0.1042–0.0071]	−0.0644 0.0958 0.5017	−0.0861 0.0383 0.0247	0.0384 0.0190 [0.0041–0.0798]	
DGLV	−0.0913 0.1259 0.4691	0.2773 0.0653 0.0000	−0.0910 0.0356 [−0.1959–−0.0279]	−0.3511 0.1294 0.0068	0.3329 0.0430 0.0000	−0.1208 0.0350 [−0.1959–−0.0573]	
Beans	−0.0944 0.1722 0.5838	0.1746 0.0794 0.0285	−0.0593 0.0337 [−0.1356–−0.0044]	0.0555 0.1148 0.6291	0.1783 0.0460 0.0001	−0.0797 0.0257 [−0.1351–−0.0351]	
Fish	−0.5430 0.1626 0.0009	0.3753 0.0874 0.0000	−0.1038 0.0468 [−0.2060–−0.0245]	−0.2694 0.1319 0.0415	0.3926 0.0476 0.0000	−0.1544 0.0396 [−0.2385–−0.0815]	
Fast Food	0.1709 0.1739 0.3264	−0.2168 0.0838 0.0101	0.0714 0.0363 [0.0123–0.1546]	*0.4186 0.1505 0.0055*	−0.3646 0.0512 0.0000	0.1362 0.0389 [0.0668–0.2220]	
MV	−0.1700 0.0976 0.0825	0.1544 0.0478 0.0014	−0.0485 0.0232 [−0.1002–−0.0115]	*−0.1126 0.0636 0.0768*	0.0725 0.0260 0.0043	−0.0319 0.0131 [−0.0606–−0.0900]	
Fish Oil	0.1903 0.1044 0.8536	0.2569 0.0533 0.0000	−0.0901 0.0335 [−0.1610–−0.0302]	−0.0377 0.0831 0.6847	0.1293 0.0334 0.0001	−0.0566 0.0182 [−0.0954–−0.0242]	
B path = −0.1682 SE = 0.0386 *p* < 0.001				B path = −0.4268 SE = 0.829 *p* = 0.0000		

HGI: high glycemic index; DGLV: dark green leafy vegetables; MV: multivitamin; Italic: trending significance.

**Table 3:** K-means clustering mean values for mature men and women.

	Men		Women		
	Western Diet	Healthy Diet and Practices	Healthy Diet	Western Diet	Dietary Practices
Exercise	−0.40543	0.55127	0.19260	−0.73570	0.29183
Breakfast	−0.224	0.30457	0.22082	−0.45985	0.05524
Whole Grain	−0.47105	0.64049	0.50487	−0.48263	−0.29798
Dairy	−0.33714	0.45842	0.54331	−0.02677	−0.67831
Caffeine	−0.17636	0.2398	0.63630	−0.18214	−0.68236
Fruits	−0.46929	0.6381	0.87216	−0.66765	−0.63118
Nuts	−0.4573	0.62179	0.71417	−0.60315	−0.47692
HGI Food	−0.01735	0.0236	0.16037	0.25840	−0.39005
Meat	−0.04312	0.05864	0.09057	0.08212	−0.17443
DGLV	−0.27635	0.37576	0.33433	−0.86512	0.19765
Beans	−0.33734	0.45869	0.23259	−0.34981	−0.04267
Fish	−0.25258	0.34343	0.21341	−0.54537	0.12310
Fast Food	0.22267	−0.30276	−0.23469	0.87862	−0.33358
MV	−0.19725	0.2682	−0.01079	−0.34881	0.26274
Fish Oil	−0.20923	0.28449	0.09741	−0.27796	0.06827
Mental Distress	0.16357	−0.22241	−0.11843	0.64137	−0.31067

HGI: high glycemic index; DGLV: dark green leafy vegetables; MV: multivitamin.

**Table 4:** Principal component analysis results for mature men and women.

Men	HD	DP	WD	Women	HD	SU	WD
Variance explained (%)	14.812	13.968	9.118	Variance explained (%)	17.477	10.775	10.364
Fruits	0.746			Fruits	0.77		
Nuts	0.702			Nuts	0.694		
Whole grain	0.612			Caffeine	0.656	−0.303	
Dairy	0.587			Dairy	0.59	−0.492	
Caffeine	0.422		0.383	Whole grain	0.586		
Fish oil		0.702		Breakfast	0.31		
MV		0.679		Beans	0.332	0.677	0.274
Fish		0.535		DGLV	0.368	0.421	−0.225
Exercise	0.388	0.509		Meat		−0.421	
Fast food		−0.39		Fish oil		0.417	
Breakfast	0.241	0.335		MV		0.347	
Mental distress	−0.214	−0.288		HGI food	0.212		0.685
Meat			0.695	Fast food	−0.209		0.583
HGI food		−0.282	0.657	Mental distress			0.523
DGLV		0.417	0.418	Exercise	0.241	0.304	−0.507
Beans	0.273		0.353	Fish	0.299	0.311	−0.336

HGI: high glycemic index; DGLV: dark green leafy vegetables; MV: multivitamin; HD: healthy diet; DP: dietary practice; WD: Western diet; SU: supplement use pattern.

**Table 5:** Spearman’s rank–order correlation results for mature women.

	Exercise	Breakfast	Whole Grain	Dairy	Caffeine	Fruits	Nuts	HGI Food	Meat	DGLV	Beans	Fish	Fast Food	MV	Fish Oil	Mental Distress
Exercise	1	0.118 **	0.200 **	−0.016	0.061	0.142 **	0.203 **	−0.151 **	−0.049	0.251 **	0.119 **	0.269 **	−0.231 **	0.095 **	0.132 **	−0.167 **
Breakfast		1	0.204 **	0.118 **	0.066	0.140 **	0.152 **	0.059	0.003	0.102 **	0.029	0.087 **	−0.099 **	0.043	0.021	−0.087 **
Whole grain			1	0.209 **	0.253 **	0.302 **	0.300 **	0.079 *	−0.027	0.167 **	0.269 **	0.164 **	−0.116 **	0.035	0.025	−0.028
Dairy				1	0.433 **	0.398 **	0.229 **	0.130 **	0.128 **	−0.043	−0.054	−0.044	0.022	−0.089 **	−0.076 *	0.061
Caffeine					1	0.430 **	0.347 **	0.034	0.110 **	0.063	0.048	0.119 **	−0.025	−0.023	0.011	0.076 *
Fruits						1	0.508 **	0.101 **	0.04	0.211 **	0.195 **	0.181 **	−0.129 **	0.008	0.042	−0.095 **
Nuts							1	0.06	−0.01	0.292 **	0.266 **	0.245 **	−0.181 **	0.042	0.080 *	−0.058
HGI food								1	0.136 **	−0.018	0.172 **	−0.097 **	0.217 **	−0.071 *	−0.04	0.151 **
Meat									1	0.074 *	−0.149 **	0.003	0.126 **	0.004	−0.104 **	−0.02
DGLV										1	0.283 **	0.287 **	−0.212 **	0.033	0.052	−0.117 **
Beans											1	0.149 **	−0.045	0.048	0.087 **	0.004
Fish												1	−0.124 **	0.101 **	0.064	−0.116 **
Fast food													1	−0.053	−0.089 **	0.160 **
MV														1	0.302 **	−0.049
Fish oil															1	−0.045
Mental distress																1

* *p* < 0.05, ** *p* < 0.01, HGI: high glycemic index; DGLV: dark green leafy vegetables; MV: multivitamin.

**Table 6:** Spearman’s rank–order correlation results for mature men.

	Exercise	Breakfast	Whole Grain	Dairy	Caffeine	Fruits	Nuts	HGI Food	Meat	DGLV	Beans	Fish	Fast Food	MV	Fish Oil	Mental Distress
Exercise	1	0.259 **	0.256 **	0.161 **	0.075	0.242 **	0.248 **	−0.165 **	0.081	0.245 **	0.095	0.226 **	−0.201 **	0.135 *	0.196 **	−0.172 **
Breakfast		1	0.227 **	0.179 **	−0.068	0.144 **	0.051	−0.078	−0.065	0.084	0.133 *	0.112 *	−0.191 **	0.134 *	0.097	0.009
Whole grain			1	0.205 **	0.185 **	0.305 **	0.350 **	0.081	−0.019	0.207 **	0.223 **	0.132 *	−0.043	0.060	0.048	−0.098
Dairy				1	0.272 **	0.348 **	0.315 **	0.096	0.099	0.048	−0.029	0.085	−0.003	0.051	0.008	−0.028
Caffeine					1	0.277 **	0.199 **	0.136 *	0.148 **	0.080	0.099	0.013	0.025	−0.059	−0.043	0.065
Fruits						1	0.470 **	0.074	−0.020	0.118 *	0.149 **	0.077	−0.135 *	0.042	0.067	−0.123 *
Nuts							1	−0.046	0.029	0.163 **	0.193 **	0.141 *	−0.046	0.032	0.102	−0.227 **
HGI food								1	0.249 **	0.032	0.159 **	−0.089	0.090	−0.061	−0.148 **	0.075
Meat									1	0.175 **	0.020	0.067	0.072	0.039	0.034	−0.023
DGLV										1	0.317 **	0.220 **	−0.156 **	0.076	0.100	−0.086
Beans											1	0.137 *	−0.081	−0.046	0.035	−0.040
Fish												1	−0.197 **	0.123 *	0.128 *	−0.189 **
Fast food													1	−0.107	−0.137 *	0.070
MV														1	0.472 **	−0.125 *
Fish Oil															1	−0.026
Mental distress																1

* *p* < 0.05, ** *p* < 0.01, HGI: high glycemic index; DGLV: dark green leafy vegetables; MV: multivitamin.

## Data Availability

The data that support the findings of this study are available from the corresponding author, upon reasonable request.

## References

[1] Begdache L., Chaar M., Sabounchi N., Kianmehr H. (2019). Assessment of Dietary Factors, Dietary Practices and Exercise on Mental Distress in Young Adults versus Matured Adults: A Cross-Sectional Study. Nutr. Neurosci..

[2] Albert P.R. (2015). Why Is Depression More Prevalent in Women?. J. Psychiatry Neurosci. Jpn..

[3] Gur R.C., Turetsky B.I., Matsui M., Yan M., Bilker W., Hughett P., Gur R.E. (1999). Sex Differences in Brain Gray and White Matter in Healthy Young Adults: Correlations with Cognitive Performance. J. Neurosci..

[4] Ingalhalikar M., Smith A., Parker D., Satterthwaite T.D., Elliott M.A., Ruparel K., Hakonarson H., Gur R.E., Gur R.C., Verma R. (2014). Sex Differences in the Structural Connectome of the Human Brain. Proc. Natl. Acad. Sci. USA.

[5] Ritchie S.J., Cox S.R., Shen X., Lombardo M.V., Reus L.M., Alloza C., Harris M.A., Alderson H.L., Hunter S., Neilson E. (2018). Sex Differences in the Adult Human Brain: Evidence from 5216 UK Biobank Participants. Cereb. Cortex.

[6] Zaidi Z.F. (2010). Gender Differences in Human Brain: A Review. Open Anat. J..

[7] Wager T.D., Phan K.L., Liberzon I., Taylor S.F. (2003). Valence, Gender, and Lateralization of Functional Brain Anatomy in Emotion: A Meta-Analysis of Findings from Neuroimaging. Neuroimage.

[8] Toga A.W., Sowell E.R., Peterson B.S., Welcome S.E., Thompson P.M., Henkenius A.L. (2003). Mapping Cortical Change across the Human Life Span. Nat. Neurosci..

[9] Cotman C.W., Berchtold N.C., Christie L.-A. (2007). Exercise Builds Brain Health: Key Roles of Growth Factor Cascades and Inflammation. Trends Neurosci..

[10] Viboolvorakul S., Patumraj S. (2014). Exercise Training Could Improve Age-Related Changes in Cerebral Blood Flow and Capillary Vascularity through the Upregulation of VEGF and ENOS. BioMed Res. Int..

[11] Miranda M., Morici J.F., Zanoni M.B., Bekinschtein P. (2019). Brain-Derived Neurotrophic Factor: A Key Molecule for Memory in the Healthy and the Pathological Brain. Front. Cell. Neurosci..

[12] Dyer A.H., Vahdatpour C., Sanfeliu A., Tropea D. (2016). The Role of Insulin-Like Growth Factor 1 (IGF-1) in Brain Development, Maturation and Neuroplasticity. Neuroscience.

[13] Ingold M., Tulliani N., Chan C.C.H., Liu K.P.Y. (2020). Cognitive Function of Older Adults Engaging in Physical Activity. BMC Geriatr..

[14] Begdache L., Marhaba R., Chaar M. (2019). Validity and Reliability of Food–Mood Questionnaire (FMQ). Nutr. Health.

[15] Craft L.L., Perna F.M. (2004). The Benefits of Exercise for the Clinically Depressed. Prim. Care Companion J. Clin. Psychiatry.

[16] Taylor C.B., Sallis J.F., Needle R. (1985). The Relation of Physical Activity and Exercise to Mental Health. Public Health Rep..

[17] Ströhle A. (2009). Physical Activity, Exercise, Depression and Anxiety Disorders. J. Neural Transm..

[18] Furukawa T.A., Kessler R.C., Slade T., Andrews G. (2003). The Performance of the K6 and K10 Screening Scales for Psychological Distress in the Australian National Survey of Mental Health and Well-Being. Psychol. Med..

[19] Krynen A.M., Osborne D., Duck I.M., Houkamau C.A., Sibley C.G. (2013). Measuring Psychological Distress in New Zealand: Item Response Properties and Demographic Differences in the Kessler-6 Screening Measure. N. Zeal. J. Psychol..

[20] Dietary Guidelines Advisory Committee (2020). Scientific Report of the 2020 Dietary Guidelines Advisory Committee: Advisory Report to the Secretary of Agriculture and the Secretary of Health and Human Services.

[21] Analysis of Total Food Intake and Composition of Individual’s Diet Based on the U.S Department of Agriculture’s 1994–96, 1998 Continuing Survey of Food Intakes by Individuals (CSFII) (2005, Final Report). https://ofmpub.epa.gov/eims/eimscomm.getfile?p_download_id=461341.

[22] Somerville L.H. (2016). Searching for Signatures of Brain Maturity: What Are We Searching for?. Neuron.

[23] Kessler R.C., Green J.G., Gruber M.J., Sampson N.A., Bromet E., Cuitan M., Furukawa T.A., Gureje O., Hinkov H., Hu C.-Y. (2011). Screening for Serious Mental Illness in the General Population with the K6 Screening Scale: Results from the WHO World Mental Health (WMH) Survey Initiative. Int. J. Methods Psychiatr. Res..

[24] Preacher K.J., Hayes A.F. (2008). Asymptotic and Resampling Strategies for Assessing and Comparing Indirect Effects in Multiple Mediator Models. Behav. Res. Methods.

[25] Abdi H., Lynne W.J. (2010). Principal Component Analysis & NBSP. Wires Comput. Stat..

[26] Schulze M.B., Hoffmann K., Kroke A., Boeing H. (2003). An Approach to Construct Simplified Measures of Dietary Patterns from Exploratory Factor Analysis. Br. J. Nutr..

[27] Begdache L., Kianmehr H., Sabounchi N., Chaar M., Marhaba J. (2018). Principal Component Analysis Identifies Differential Gender-Specific Dietary Patterns That May Be Linked to Mental Distress in Human Adults. Nutr. Neurosci..

[28] Kaplan B.J., Crawford S.G., Field C.J., Simpson J.S.A. (2007). Vitamins, Minerals, and Mood. Psychol. Bull..

[29] Chalon S. (2006). Omega-3 Fatty Acids and Monoamine Neurotransmission. Prostaglandins Leukot. Essent. Fat. Acids.

[30] Silva R.V., Oliveira J.T., Santos B.L.R., Dias F.C., Martinez A.M.B., Lima C.K.F., Miranda A.L.P. (2017). Long-Chain Omega-3 Fatty Acids Supplementation Accelerates Nerve Regeneration and Prevents Neuropathic Pain Behavior in Mice. Front. Pharmacol..

[31] Atlantis E., Chow C.-M., Kirby A., Singh M.F. (2004). An Effective Exercise-Based Intervention for Improving Mental Health and Quality of Life Measures: A Randomized Controlled Trial. Prev. Med..

[32] Benton D. (2002). Carbohydrate Ingestion, Blood Glucose and Mood. Neurosc. Biobehav. Rev..

[33] Bourre J.M. (2005). Dietary Omega-3 Fatty Acids and Psychiatry: Mood, Behaviour, Stress, Depression, Dementia and Aging. J. Nutr. Health Aging.

[34] Fernstrom J.D., Fernstrom M.H. (2007). Tyrosine, Phenylalanine, and Catecholamine Synthesis and Function in the Brain. J. Nutr..

[35] Nicholson S.A. (1989). Stimulatory Effect of Caffeine on the Hypothalamo-Pituitary-Adrenocortical Axis in the Rat. J. Endocrinol..

[36] Kaster M.P., Machado N.J., Silva H.B., Nunes A., Ardais A.P., Santana M., Baqi Y., Müller C.E., Rodrigues A.L.S., Porciúncula L.O. (2015). Caffeine Acts through Neuronal Adenosine A2A Receptors to Prevent Mood and Memory Dysfunction Triggered by Chronic Stress. Proc. Natl. Acad. Sci. USA.

[37] Richards G., Smith A. (2015). Caffeine Consumption and Self-Assessed Stress, Anxiety, and Depression in Secondary School Children. J. Psychopharmacol..

[38] Daniel P.M., Love E.R., Moorhouse S.R., Pratt O.E. (1981). The Effect of Insulin upon the Influx of Tryptophan into the Brain of the Rabbit. J. Physiol..

[39] Tagliamonte A., DeMontis M.G., Olianas M., Onali P.L., Gessa G.L. (1976). Possible Role of Insulin in the Transport of Tyrosine and Tryptophan from Blood to Brain. Adv. Exp. Med. Biol..

[40] Nishizawa S., Benkelfat C., Young S.N., Leyton M., Mzengeza S., Montigny C.D., Blier P., Diksic M. (1997). Differences between Males and Females in Rates of Serotonin Synthesis in Human Brain. Proc. Natl. Acad. Sci. USA.

[41] Jacka F.N., Pasco J.A., Mykletun A., Williams L.J., Hodge A.M., O’Reilly S.L., Nicholson G.C., Kotowicz M.A., Berk M. (2010). Association of Western and Traditional Diets with Depression and Anxiety in Women. Am. J. Psychiatry.

[42] Wurtman R.J., O’Rourke D., Wurtman J.J. (1989). Nutrient Imbalances in Depressive Disorders. Possible Brain Mechanisms. Ann. N. Y. Acad. Sci..

[43] Hariri N., Gougeon R., Thibault L. (2010). A Highly Saturated Fat-Rich Diet Is More Obesogenic than Diets with Lower Saturated Fat Content. Nutr. Res..

[44] Begdache L., Sadeghzadeh S., Derose G., Abrams C. (2021). Diet, Exercise, Lifestyle, and Mental Distress among Young and Mature Men and Women: A Repeated Cross-Sectional Study. Nutrients.

[45] Lautenschlager N.T., Almeida O.P., Flicker L., Janca A. (2004). Can Physical Activity Improve the Mental Health of Older Adults?. Ann. Gen. Hosp. Psychiatry.

[46] Gariballa S., Alessa A. (2020). Associations between Low Muscle Mass, Blood-Borne Nutritional Status and Mental Health in Older Patients. BMC Nutr..

